# Information on pros and cons of prostate-specific antigen testing to men prior to blood draw: A study from the National Prostate Cancer Register (NPCR) of Sweden

**DOI:** 10.3109/00365599.2012.691110

**Published:** 2012-05-31

**Authors:** Jon Fridriksson, Katarina Gunseus, Pär Stattin

**Affiliations:** 1 Department of Surgical and Perioperative Sciences, Urology and Andrology, Umeå University Hospital, Umeå; 2 Department of Surgery, Urology Service, Memorial Sloan-Kettering Cancer Center, New York City, USA

**Keywords:** decision aids, guidelines, prostate cancer, prostate-specific antigen

## Abstract

*Objective*. Recent guidelines on serum testing of prostate-specific antigen (PSA) levels in asymptomatic men emphasize the importance of an informed decision. This study assessed the proportion of men who had received written or oral information on the possible consequences of testing of serum levels of PSA before blood draw. Material and methods. From the National Prostate Cancer Register (NPCR) in Sweden, 600 men per year were randomly selected out of all men with T1c prostate cancer who were diagnosed in the work-up of a PSA test as a part of health examination in 2006–2008. In a mailed questionnaire these men were asked whether and how they had been informed about the pros and cons of a PSA test prior to blood draw. Results. In total, 1621 out of 1800 men (90.1%) responded to the questionnaire; 39/1563 (2.5%) reported that they had received only written information before testing, 179/1563 (11.5%) had received both oral and written information, 763/1563 (48.8%) had received oral information only, 423/1563 (27.1%) had not received any information and 159/1563 (10.2%) were not aware of that a PSA test had been performed. Conclusions. The proportion of men who had received written information on the pros and cons of a PSA test before blood draw in the setting of a health examination was low. Improved routines for giving information to the patient before a PSA test are warranted.

## Introduction

The pros and cons of prostate cancer screening are the subject of much debate, and there is disagreement on the use of serum prostate-specific antigen (PSA) testing [1]. Recently, the United States Preventive Services Task Force published a Draft Recommendation Statement in which they concluded with moderate certainty that PSA-based screening for prostate cancer has no net benefit [2,3]. Furthermore, according to the European Association of Urology (EAU) guidelines, there is currently no evidence for introducing widespread population-based screening for the early detection of prostate cancer in all men [4]. However, screening for prostate cancer may advance diagnosis by at least 10 years [5].

The European Randomized Study of Screening for Prostate Cancer (ERSPC) and the Göteborg Screening Study showed that screening and early treatment decreased prostate cancer-specific mortality by 20–44%, with an absolute risk reduction of 0.07% and 0.5% [6,7]. However, it has also been suggested that 23–42% of PSA-detected cancers are overdiagnosed [5].

Although the most widely accepted guidelines recommend against screening for prostate cancer, a man may still choose to be screened because he places a higher value on the possibility of benefit, however small, than on the known drawbacks that accompany the screening and treatment of screening-detected prostate cancer, particularly the harms of overdiag-nosis and overtreatment. In view of this controversy, there is consensus that men need to be well informed before they undergo a PSA test. This can be achieved by use of decision aids that provide balanced, evidence-based information on the pros and cons of PSA-based screening for prostate cancer. Such decision aids have been shown to increase knowledge about prostate cancer screening [8–10], decrease participation in screening and reduce the uptake of PSA testing [10–12]. There is, to the authors’ knowledge, limited information on the proportion of men who had received information on the pros and cons of the PSA test before blood draw, a first step to an informed decision.

The aim of this study was to assess the proportion of men subsequently diagnosed with prostate cancer who had received information prior to blood draw.

## Material and methods

### Study population and data collection

The National Prostate Cancer Register (NPCR) of Sweden started in 1996 and captures more than 96% of all newly diagnosed prostate cancer as compared to the Swedish Cancer Registry (SCR) [13,14], to which registration is compulsory and mandated by law. The capture rate of the SCR has been reported to be virtually complete, at approximately 98% for solid tumours in patients younger than 75 years [15]. The NPCR includes the date of diagnosis, age at diagnosis, tumour stage, tumour differentiation, serum level of PSA at time of diagnosis, and primary treatment given or planned up to 6 months after date of diagnosis. A more detailed description of NPCR is given elsewhere [16,17].

In 2007 National Guidelines on detection, diagnostic work-up and treatment of prostate cancer were issued by the National Board of Health and Welfare in Sweden [18]. These guidelines strongly recommended that, in order to make an informed decision on PSA testing, all asymptomatic men considering PSA testing should receive written information on the pros and cons of the PSA test prior to the blood draw. To that end, a brochure with patient information was published [19].

In the years 2006–2008, 600 men were annually randomly selected out of all men with T1c prostate cancer in the NPCR who were diagnosed in the work-up of a PSA test as a part of health examination according to the registration in the NPCR. The accuracy of tumour staging for prostate cancer in the NPCR is 81.0% and the total number of prostate cancer cases registered in the NPCR was 9111 in 2006, 8853 in 2007 and 8788 in 2008 [17]. The mean age of the men in this study was 64.2 (range 40–88) years. In a questionnaire, sent out by ordinary mail, these randomly selected men with screen-detected prostate cancer were asked whether they had been informed about the pros and cons of the PSA test before the blood draw and how they had received this information. Furthermore, the men were asked whether and how they had been informed about their PSA level. The questionnaire was sent out 2–3 years after the diagnosis of prostate cancer, as it can take up to 2 years before all patients are entered in the register. For men diagnosed in 2006 the questionnaires were sent out in March 2009, for men diagnosed in 2007 in April 2010 and for men diagnosed in 2008 they were sent out in October 2010. A reminder was sent to non-responders 2–3 months after the first letter. The survey questionnaire included the four questions shown in Table I.

**Table I:** Questionnaire used in this study with the ratio of reported answers.

	2006	2007	2008	Total
What was the reason for your first PSA test?	*n* = 538 (%)	*n* = 534 (%)	*n* = 537 (%)	*n* = 1609 (%)
I had voiding symptoms	64(11.9)	71(13.3)	75(14.0)	210 (13.1)
I had other symptoms from the urineorgenital organs	18(3.3)	15(2.8)	18(3.4)	51(3.2)
I was worried about having prostate cancer	95(17.7)	94(17.6)	104 (19.4)	293 (18.2)
I was worried about having prostate cancer as I have relatives diagnosed with prostate cancer	94(17.5)	79(14.8)	102 (19.0)	275 (17.1)
My wife/girlfriend/partner urgedme	87(16.2)	76(14.2)	74(13.8)	237 (14.7)
My doctor recommended that I should have the PSA test	131 (24.3)	162 (30.3)	152 (28.3)	445 (27.7)
Discussion and media “you should check your PSA”	113 (21.0)	122 (22.8)	119 (22.2)	354 (22.0)
I had sought medical care for something else	59(11.0)	62(11.6)	63(11.7)	184 (11.4)
I hadaregular health examination	252 (46.8)	234 (43.8)	267 (49.7)	753 (46.8)
What information did you receive before the PSA test?	*n* = 521 (%)	*n* = 520 (%)	*n* = 522 (%)	*n* = 1563 (%)
Oral information on the pros and cons of PSA testing	253 (48.6)	263 (50.6)	247 (47.3)	763 (48.8)
Oral and written information on the pros and cons of PSA testing	51 (9.8)	55 (10.6)	73 (14.0)	179 (11.5)
Written informationon the pros and cons of PSA testing	10(1.9)	17(3.3)	12 (2.3)	39(2.5)
No information on the pros and cons of PSA testing	144 (27.6)	144 (27.7)	135 (25.9)	423 (27.1)
No information that the PSA test had been done	63(12.1)	41(7.9)	55(10.5)	159 (10.2)
Did the informationonthe pros and consof PSA testing influence your decision on having the test?	*n* = 520 (%)	*n* = 520 (%)	*n* = 526 (%)	*n* = 1566 (%)
No	267 (51.3)	306 (58.8)	281 (53.4)	854 (54.5)
Yes	94(18.1)	82(15.8)	92(17.5)	268 (17.1)
No information on the pros and cons of PSA testing	158 (30.4)	132 (25.4)	152 (28.9)	442 (28.2)
How did you receive information on the result from the PSA test?	*n* = 539 (%)	*n* = 531 (%)	*n* = 533 (%)	*n* = 1603 (%)
At an appointment with my physician	329 (61.0)	342 (64.4)	342 (64.2)	1013 (63.2)
With a telephone call	97(18.0)	95(17.9)	98(18.4)	290 (18.1)
By a letter 107	(19.9)	84(15.8)	90(16.9)	281 (17.5)
I was not informed on the PSA value	6(1.1)	10(1.9)	3(0.6)	19(1.2)

PSA = prostate-specific antigen.

### Ethical approval

The Research Ethics Review Board of Umeå University Hospital reviewed the study protocol and considered that it did not need approval.

## Results

In total, 1621 out of 1800 men (90.1%) responded to the questionnaire (Table I). The response rate was stable over time: 545 out of 600 (90.8%) of men diagnosed in 2006, 535 out of 600 (89.2%) in 2007 and 541 out of 600 (90.2%) in 2008.

For almost half of the men PSA testing was a part of a health examination, approximately 27.7% reported that their physician had recommended the test and 22.0% of the men reported that they had had a PSA test because they had received information about it from the media.

**Figure 1:**
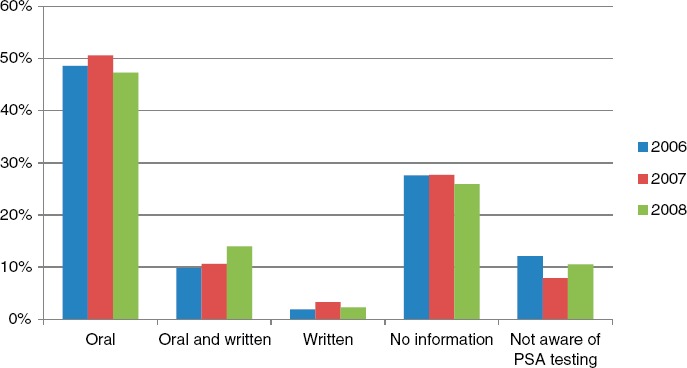
Ratio of men receiving information on prostate-specific antigen (PSA) test (oral, oral and written, or written information) and those not receiving information or not aware of PSA testing, stratified by calendar time.

**Figure 2:**
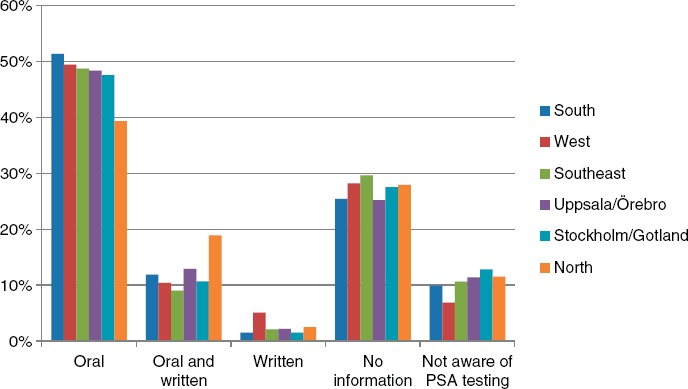
Ratio of men receiving information on prostate-specific antigen (PSA) testing in the six different healthcare regions.

Of the responders, 62.8% reported that they had received information before testing, 27.1% had not received information and 10.2% were not aware of that a PSA test had been performed. There was little difference in the proportion of men informed during 2006–2008. Of the men who received information on PSA testing, 48.8% had been informed orally, 11.5% had received both written and oral information, and 2.5% had received only written information. There was a small increase over time in the ratio of men who had received written or both oral and written information, from 11.7% in 2006 to 13.9% in 2007 and 16.3% in 2008 (Figure 1). There were also small differences in the proportion of men who had received information in the different healthcare regions in Sweden (Figure 2). In total, 268 out of 1566 (17.1%) men reported that the information they received had affected their decision on taking the PSA test. Almost all men had been informed about their PSA level, most often at an appointment with their physician, but some had received the information over the telephone or by a letter.

## Discussion

Only 14.0% of men in this survey recalled that they had received written or both oral and written information about the pros and cons of PSA testing prior to blood draw. There was only a small increase in the proportion of men who had received written or both written and oral information after the National Guidelines had been issued and virtually no change in the proportion of men who had received information in any way. Interestingly, 22.0% of the men reported receiving information about PSA testing from the media, a higher proportion than those who received written information from the healthcare provider. Somewhat disturbingly, 10.2% of the men were not aware that they had undergone PSA testing.

The main strength of this study is the high response rate (90.1%) to the mail questionnaire and the high capture rate of the NPCR that was used in the study. The main drawback of the study is that only men with prostate cancer were included, which limits the applicability of the results. Another limitation is that the results are susceptible to recall bias as there was a long time between the PSA testing and data collection.

Earlier studies have shown that men have little knowledge about prostate cancer [20,21], implying that men need decision support regarding PSA-based screening. Furthermore, the findings of this study are in accordance with other studies suggesting that men need to be better informed about the pros and cons of PSA-based screening for prostate cancer. In a recent questionnaire survey from the USA, physicians were asked about prostate cancer screening practices. The study include 555 subjects, of whom 69% responded, and 74% of the responders reported that they discussed the risks and benefits of PSA testing with the patient before blood draw but very few (10%) used any written information to support the patient's decision concerning testing [22]. In a telephone survey from the USA, including 375 men, about 70% of subjects reported that they had discussed PSA screening for prostate cancer with a healthcare provider before making a decision about PSA testing, including 14% who did not subsequently undergo testing. Furthermore, almost all subjects in that study reported discussing the pros of PSA testing, although substantially fewer reported discussing the cons of testing and only 21% reported discussing both pros and cons of PSA testing and being asked their preference for testing [21].

Providing men with balanced, evidence-based information about the pros and cons of PSA testing is necessary but not sufficient to enhance informed decision making, as not all men use the decision aids. Partin et al. found that only half of men receiving a prostate cancer screening decision aid, prior to a general internal medicine appointment, reported using the aid [23], indicating that substantial effort is needed to improve the use of decision aids.

The present study group consisted of men living in Sweden and a response was obtained from 90.1% of the randomly selected men with prostate cancer who, according to the NPCR, were detected in the workup of an elevated PSA detected at a health check-up. It is difficult to assess the extent to which these results on information provided to asymptomatic men before PSA testing are generalisable to other populations. The type of healthcare system will influence both how information is conveyed and the incentives to carry out the test. Attitudes regarding the benefits of screening can also differ substantially between populations. However, studies from the USA have reported a very similar proportion of men who had received information prior to PSA testing [21,22].

In summary, there is consensus that men need to be well informed before a PSA test on the consequences of the test, but this study indicates that in clinical day-to-day practice the proportion of men who receive written information on the pros and cons of a PSA test prior to blood draw is low. Improvements in the distribution of information and the use of existing decision aids are warranted.
